# Contraction of Entangled Polymers After Large Step Shear Deformations in Slip-Link Simulations

**DOI:** 10.3390/polym11020370

**Published:** 2019-02-20

**Authors:** Yuichi Masubuchi

**Affiliations:** Department of Materials Physics, Nagoya University, Nagoya 4648603, Japan; mas@mp.pse.nagoya-u.ac.jp

**Keywords:** entangled polymers, coarse-grained simulation, viscoelasticity, rheology

## Abstract

Although the tube framework has achieved remarkable success to describe entangled polymer dynamics, the chain motion assumed in tube theories is still a matter of discussion. Recently, Xu et al. [ACS Macro Lett. 2018, 7, 190–195] performed a molecular dynamics simulation for entangled bead-spring chains under a step uniaxial deformation and reported that the relaxation of gyration radii cannot be reproduced by the elaborated single-chain tube model called GLaMM. On the basis of this result, they criticized the tube framework, in which it is assumed that the chain contraction occurs after the deformation before the orientational relaxation. In the present study, as a test of their argument, two different slip-link simulations developed by Doi and Takimoto and by Masubuchi et al. were performed and compared to the results of Xu et al. In spite of the modeling being based on the tube framework, the slip-link simulations excellently reproduced the bead-spring simulation result. Besides, the chain contraction was observed in the simulations as with the tube picture. The obtained results imply that the bead-spring results are within the scope of the tube framework whereas the failure of the GLaMM model is possibly due to the homogeneous assumption along the chain for the fluctuations induced by convective constraint release.

## 1. Introduction

For the description of the viscoelasticity of polymers, the tube model has achieved remarkable success [1,2,3]. In this framework, entangled polymer dynamics are replaced by the dynamics of a single chain confined in a tube-shaped constraint fixed in space [4,5,6]. Under the quiescent state, the chain slides back and forth randomly in the tube, and the end portion that protrudes from the tube edge loses the orientational memory. This chain dynamic is called reptation, and the predictions derived from the reptation picture for the diffusion and the relaxation time are qualitatively consistent with experiments.

The tube picture works well for the description of non-linear viscoelasticity, such as non-linear relaxation modulus under large step deformations [7,8,9]. At the application of deformation, the tube is assumed to be affinely deformed. The relaxation of the system starts with the contraction of the chain inside the tube. Afterward, reptation occurs to induce the orientational relaxation. This two-step relaxation picture has been widely accepted because of the success of the tube model proposed by Doi and Edwards [7,8,9]. In particular, the theory is consistent with the experimentally observed time-strain separability for relaxation modulus under large step shear deformations [10]. Further, the damping function is surprisingly well-described by the amount of annihilated tube portion due to the chain contraction. From the tube picture, several constitutive equations have been proposed and the predictions they make are in semi-quantitative agreement with experimental data [11,12,13,14]. Attempts have also been made for coarse-grained molecular simulations, in which the polymers move under dynamical constraints similar to the tube [15,16,17,18].

**Figure 1:**
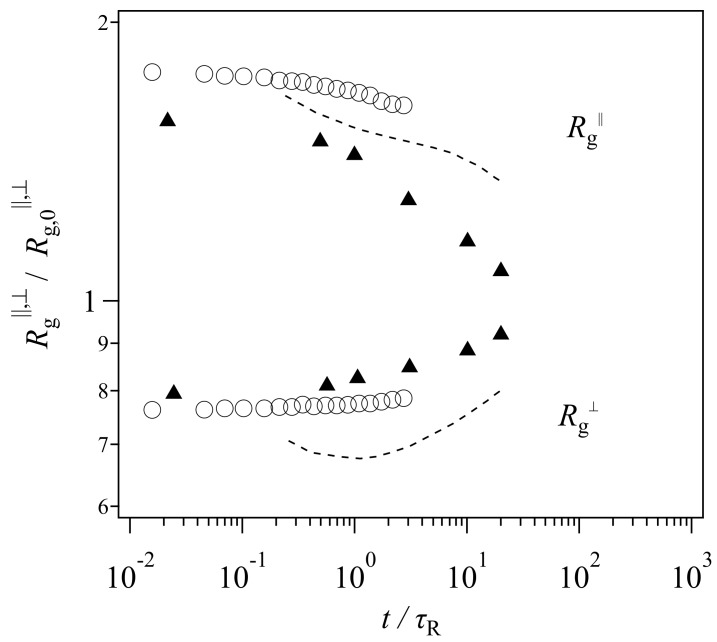
Time development of gyration radius parallel (**top**) and perpendicular (**bottom**) to the stretching direction denoted as $R_{g}^{∥}$ and $R_{g}^{⊥}$, respectively. The gyration radius and time are normalized by the equilibrium value and the Rouse time, respectively. Circles and triangles show the data from a bead-spring simulation [19] and an experiment [20]. The dashed curve indicates the prediction by the GLaMM model. The data are extracted from Xu et al. [19].

Because the tube picture is a working hypothesis, it has been exposed to critical tests. For example, recently Xu et al. [19] performed bead-spring simulations for a reasonably entangled melt (with a bead number per chain of 2000) under a step uniaxial deformation. They observed the time-development of gyration radii and compared it with a tube model, the so-called GLaMM model [13]. The extracted data are shown in Figure 1. Note that the range of vertical and horizontal axes is different from the original presentation made by Xu et al. [19], who employed different plot ranges for each model to emphasize qualitative consistency. Nevertheless, their results demonstrate that the gyration radius perpendicular to the elongated direction $R_{g}^{⊥}$ monotonically increases with time in the simulation (circle), whereas that in the examined tube model shows non-monotonic behavior. See the dashed curve, which decreases in the short time regime before the Rouse relaxation time followed by an increase in the longer time period.

Based on this comparison and the experimental results obtained from SANS [20] (shown by triangles in Figure 1), Xu et al. [19] assessed that the two-step relaxation mechanism proposed in the tube picture was not adequate, and that a new molecular view was necessary. However, Figure 1 demonstrates that the magnitude of the discrepancy of the experimental data is not that different between the bead-spring simulation and the GLaMM model. Besides, the comparison was made for one specific tube model. Although the model includes the relevant relaxation mechanisms, i.e., reptation, chain stretch, contour length fluctuations, and thermal and convective constraint release, the mathematical implementation is arbitrary. Specifically, although the GLaMM model has been well-examined for start-up shear deformations, Cheng et al. [21] have shown that the GLaMM model is not compatible with experiments for non-linear relaxation modulus obtained under large step shear and uniaxial deformations. Note that Xu et al. [19] only showed the comparison for the chain conformation, however, they did not present the GLaMM prediction for the stress relaxation. Thus, the failure of the GLaMM model for the chain conformation might not be due to the conventional tube scenario of the chain relaxation, rather, it may be due to the implementation of the relaxation mechanisms for the model. 

In this study, the chain contraction after step elongation was examined by two different slip-link simulations that have been separately developed by Doi and Takimoto [18], and Masubuchi et al. [17] Hereafter, the model by Doi and Takimoto is referred to as the DT model, whereas that by Masubuchi et al. is called the primitive chain network (PCN) model. Both models are constructed following the reptation picture and consider the essential relaxation mechanisms, as with the GLaMM model. However, the implementation of the constraint release (CR) is different. Namely, due to the nature of the single chain modeling in the GLaMM model, CR is considered in a mean-field manner whereas in the slip-link models, CR occurs as a result of the destruction and creation of slip-links between different chains in the system. Due to the difference in the CR implementation, the prediction of chain conformation is model dependent. Indeed, the results demonstrated that DT and PCN simulations quantitatively reproduced the bead-spring simulations of Xu et al. Details are shown below.

## 2. Models and Simulations

**Figure 2:**
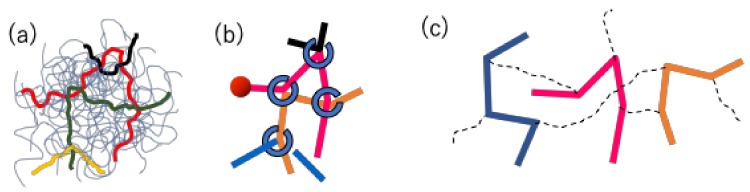
Schematics of the slip-link models used in this study. (**a**) An entangled polymer network, (**b**) a slip-link network considered in the PCN model, and (**c**) a group of chains considered in the DT model. In Figure (**c**), dotted curves indicate the pairing of slip-links.

Because the details for both models have already been published [17,18,22], only brief descriptions are given below. The schematics of the models are shown in Figure 2. In both models, a group of entangled polymers is replaced by a slip-link network that consists of network nodes, strands, and dangling ends. Each polymer chain is represented by a path connecting two dangling ends through the strands and nodes. At each node, two chains are bundled by a slip-link in a pair. 

For the PCN model, the dynamics considered include the Brownian motion of network nodes, the chain sliding at the nodes, and the creation and destruction of the nodes at the chain ends. The position of network nodes obeys the Langevin-type equation of motion, in which the force balance is considered among the drag-force, strand tension, osmotic force, and random force. The chain sliding is described by the rate equation for the Kuhn segment number on each strand, and in the equation, the same force balance with the node position is considered between the connected segments along the chain. When the number of Kuhn segments at a dangling end exceeds a specific critical value as a result of the dynamics mentioned above, a new slip-link (i.e., a new network node) is created on the end segment by hooking another segment, which is randomly chosen from the surrounding segments within a specific distance. Vice versa, when the number of Kuhn segments becomes smaller than the other critical value, the slip-link connected to the end segment is removed, and the paired chain is released. 

For the DT model, the connection between chains in real-space is not considered. Namely, the chains are separately managed from each other as shown in Figure 2c. The pairing between chains via slip-links is maintained in a book-keeping manner. However, the distance between chains in real space is not considered for the pairing. Due to this model construction, the force balance around entanglement is not considered. Indeed, under a deformation, the slip-link position and the segment vector change affinely, and no relaxation takes place except the reptation of the chain and CR by the partner chain. 

In the model setup mentioned above, the established relaxation mechanisms are naturally considered for both models. Namely, the chain obeys reptation motion by the creation/destruction of slip-links at the chain ends. The slip-link dynamics also induces the constraint release for the paired chain. For PCN, contour length fluctuation occurs as a result of the local force balance for both the node position and the chain sliding, whereas for DT, it is directly implemented by the fluctuations of the segment length at the chain ends, and is only concerned with the longest Rouse mode. The chain stretch is also straightforwardly implemented.

The simulations were made to reproduce the results found by Xu et al. [19] for the melt of bead-spring chains with a bead number per chain of 2000 and a bead number density of 0.85. According to previous studies [23,24], this bead-spring simulation is comparable to a PCN simulation with an average segment number per chain of 50. Before the uniaxial deformation, the system was sufficiently equilibrated with the simulation box size of 15^3^, and the average segment number density was 10. Here, the unit of length is the average segment length under equilibrium. For DT simulation, the segment number of 33 per chain was used for consistency with the GLaMM calculations by Xu et al. The number of chains calculated was 10,000. As with PCN simulations, sufficient equilibration was attained before the deformation. For both simulations, the relaxation after a step uniaxial deformation with a Hency strain of 0.587 was observed, as performed by Xu et al. (Note that a Hency strain of 0.587 is comparable to a stretching ratio of 1.8.) For the statistics, eight independent simulation runs with different initial configurations were made, and the results were ensemble averaged. 

## 3. Results and Discussion

**Figure 3:**
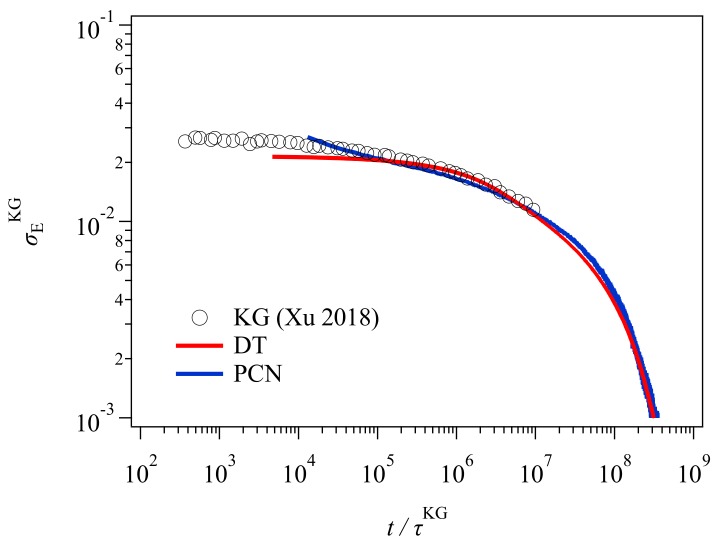
Stress relaxation after step uniaxial deformation with a Hency strain of 0.587. Circles indicates the data of Xu et al. for the Kremer-Grest (KG) simulation. Red and blue solid curves are the results from the DT and PCN simulations, respectively. The results from DT and PCN are converted to KG units via the conversion factors shown in the text.

Figure 3 shows the stress relaxation after the uniaxial deformation. Here, for conversion of time and stress, the conversion factors previously obtained [23,24] were used for the PCN result. For the DT result, the conversion factors were obtained from the comparison to the PCN result as explained in the Appendix A. The conversion relationships used for time and stress (modulus) are as follows.

(1) $$1.3\times 10^{4}\tau^{\mathrm{PCN}}=4.6\times 10^{3}\tau^{\mathrm{DT}}=\tau^{\mathrm{KG}}$$

(2) $$1.9\times 10^{-2}G^{\mathrm{PCN}}=3.2\times 10^{-2}G^{\mathrm{DT}}=G^{\mathrm{KG}}$$

The superscript denotes the model, and KG stands for Kremer and Grest [25], who are the founders of the bead-spring simulations. $\tau^{\mathrm{KG}}$ and $G^{\mathrm{KG}}$ are the unit of time and stress for Lennard-Jones liquids denoted by $\tau^{\mathrm{KG}}=\sigma \sqrt{m/\epsilon }$ and $G^{\mathrm{KG}}=\epsilon /\sigma^{3}$, respectively. Here, m is the bead mass, and σ and ϵ are the parameters for the Lennard-Jones potential. For the conversion relationships shown above, for the DT model, the unit of time and stress correspond to the Rouse time of one tube segment $\tau_{e}$ and the plateau modulus $G_{e}$, respectively. Xu et al. [19] reported these values for their GLaMM calculations as $\tau^{\mathrm{KG}}=3.3\times 10^{3}\tau^{\mathrm{GLaMM}}$ and $G^{\mathrm{KG}}=1.4\times 10^{-2}G^{\mathrm{GLaMM}}$. These values are somewhat different from those in Equations (1) and (2), possibly due to the difference in implementation for the relaxation mechanisms. For the PCN model, the difference is due to the imposed fluctuations around the entanglements [26]. Nevertheless, the results from the three different simulations nicely overlap, except in the short time regime for $t/\tau_{\mathrm{KG}}\leq 10^{5}$, where the higher Rouse modes and the glassy modes come into play in the KG result, whereas DT and PCN do not consider those modes. The other possible reason would be the application of initial step deformation, for which the strain rate was infinite for DT and PCN whereas it was finite for Xu et al. [19].

**Figure 4:**
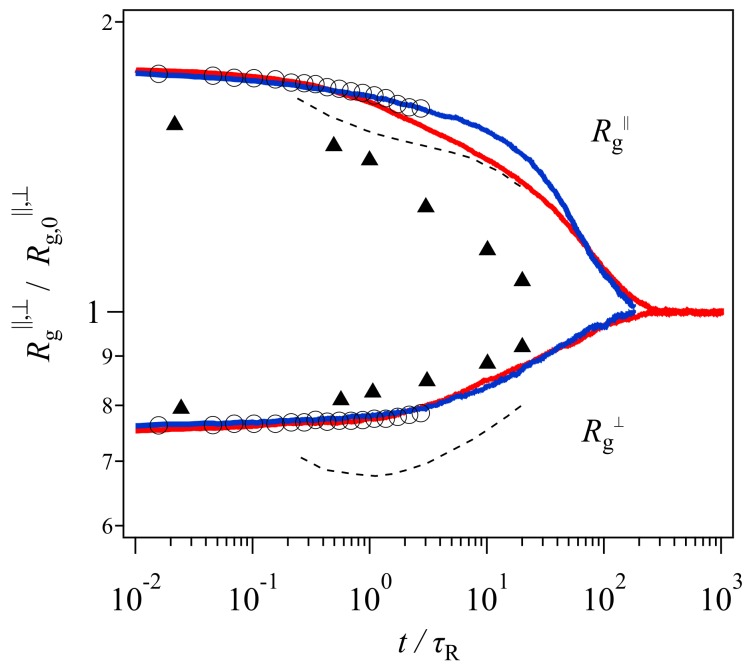
Time development of gyration radius parallel (**top**) and perpendicular (**bottom**) to the stretching direction. The gyration radius and time are normalized by the equilibrium value and the Rouse time, respectively. Circles and triangles show the data from the bead-spring simulations and the experiment. The dashed curve indicates the prediction by the GLaMM model. These data were extracted from the paper by Xu et al. [19] Red and blue curves are the results from the DT and PCN simulations, respectively.

Figure 4 shows the gyration radius with the data of Xu et al. [19] shown in Figure 1 for comparison. Here, the Rouse relaxation time used for the normalization of time is $\tau_{R}=3.7\times 10^{6}\tau_{\mathrm{KG}}$. The DT and PCN results (red and blue curves) are in excellent agreement with the bead-spring simulation (circle), and discrepant from the GLaMM prediction (dashed curve) within the time range in which the bead-spring data were available. Beyond $\tau_{R}$, for $R_{g}^{∥}$, PCN (blue) shows a slower relaxation than DT (red). This retarded relaxation in PCN is related to the difference in the chain contraction discussed later. Nevertheless, the results shown in Figure 4 unequivocally indicate that the failure of the GLaMM prediction is not due to the tube picture, but possibly due to the difference in the mathematical implementation of the relaxation mechanisms. Indeed, all three coarse-grained models follow the tube framework. 

In contrast, the experimental data (triangle) cannot be captured, although $R_{g}^{⊥}$ is somewhat better predicted than $R_{g}^{∥}$. This discrepancy is possibly due to the failure in the modeling of this specific experiment. For example, there is a difference in the deformation conditions between the simulations and the experiment. In the simulations, owing to the periodic boundary condition, the application of step uniaxial deformation is straightforward and the strain is retained during the relaxation. In the experiment compared here, after the stretching, the position of both ends of the specimen was kept whereas the strain applied to the material probably increased in time due to the flow induced by gravity, as reported earlier [27]. An unavoidable relaxation during the quenching for neutron measurements is the other possible reason. 

For the GLaMM model, the failure could be due to the implementation of constraint release in the single chain modeling. In the GLaMM theory, the number of tube segments is constant with time, and the segment position always fluctuates with time. The magnitude of fluctuations for the segment position is uniform along the chain, and it describes the effects of thermal and convective constraint release in a mean-field manner. Although Graham et al. [13] assumed that fluctuations are enhanced when the contraction rate is high, the minimum observed for $R_{g}^{⊥}$ suggests that the fluctuations to the perpendicular direction are not sufficient. For the DT and PCN models, the fundamental picture for the chain dynamics is the same as that considered in the GLaMM model. However, these slip-link models do not employ single chain modeling and mean-field approximations. Consequently, the distribution of entanglement along the chain is not uniform, and the fluctuations induced by constraint release are not uniform as well. Besides, as shown below, the number of entanglements per chain changes with time. These inhomogeneities add other fluctuations that possibly remove the minimum in $R_{g}^{⊥}$. Finally, note that in the original paper by Graham et al. [13], they evaluated their theory only for the constitutive model derived from the molecular model, although they presented the equation of motion for segments. The conformational dynamics discussed here were calculated by Xu et al. [19], who made the code for the calculations. 

**Figure 5:**
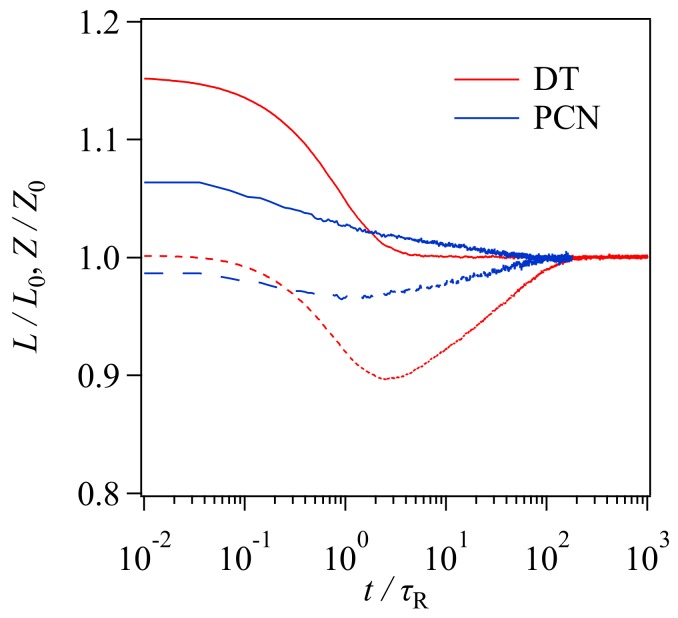
Time development of entanglement density Z (**solid curves**), and tube contour length L (**dashed curves**) for DT (**red curves**) and PCN (**blue curves**) simulations. Z and L are normalized by the equilibrium values.

Although the gyration radius does not show the non-monotonic behavior in the DT and PCN simulations, the chain contraction assumed in the tube picture is observed as demonstrated in Figure 5, in which the tube contour length and the entanglement density are seen. For the DT simulation, the tube contour (solid red curve) clearly shows the contraction, as it decays with the characteristic relaxation time identical to the Rouse time. Due to this chain contraction, the entanglement density decreases (dashed red curve). After the chain contraction, the entanglement density revives to the equilibrium value via the reptation motion with the longest relaxation time. These chain dynamics are the same as those assumed in the tube framework [7]. The chain contraction occurs for PCN as well (see solid blue curve). However, the relaxation occurs in a two-step manner, as reported earlier by Furuichi et al. [28,29,30,31]. For the two relaxation processes, the characteristic time is close to the Rouse time and the longest relaxation time, respectively. The long-time contraction in PCN induces the difference in $R_{g}^{∥}$ shown in Figure 4, in which $R_{g}^{∥}$ for PCN is retarded to that for DT in $t<\tau_{R}$. The origin of this slow relaxation is the propagation of orientational relaxation via the force balance around entanglement. The entanglement density (dashed blue curve) decreases due to the chain contraction, and it shows a minimum around the Rouse time as with DT. However, the magnitude of the decrease is much smaller. These differences between the models are not seen in the gyration radii, as with the stress shown in Figure 3 and Figure 4. This result implies that the gyration radius is not strongly sensitive to the difference in molecular motion, such as chain contraction and loss of entanglement. 

## 4. Conclusions

In this study, the slip-link simulations were performed and compared to the bead-spring simulation by Xu et al. [19] for the relaxation of stress and gyration radii of entangled polymers after the step uniaxial deformation. The slip-link simulations excellently reproduced the bead-spring results, which were not captured by the tube model known as the GLaMM model. The results suggest that the bead-spring results can be explained with the tube framework, but the implementation of the molecular picture induces the failure of the GLaMM model. The differences among the models imply that the homogeneous assumption for the fluctuations along the chain in the GLaMM model may be the reason. Note however, that further investigations are necessary using various initial strains and deformations. 

## Figures and Tables

**Figure A1:**
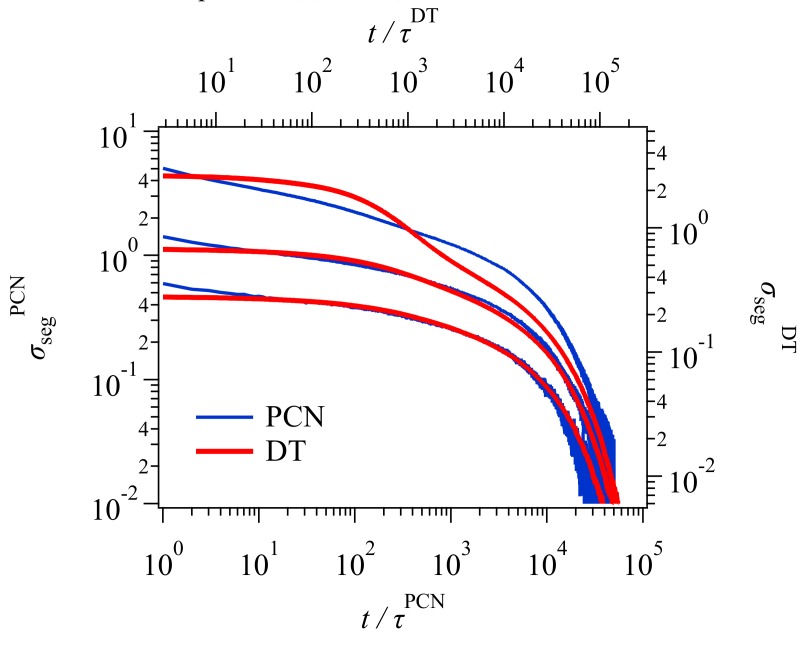
Stress relaxation after step uniaxial deformations with a Hency strain of 0.3, 0.588 and 1.2 from bottom to top. Red and blue curves show the results for the DT and PCN simulations, respectively. The stress is normalized by the entanglement segment density under equilibrium.

## Appendix A

In this section, determination of the conversion parameters for DT model is discussed. In principle, units of stress (modulus) and time can be unequivocally determined by fitting for relaxation modulus. However, in practice, the fitting is an arbitrary matter for a single curve. For this reason, the stress relaxation was calculated with various initial strains as shown in Figure A1. Interestingly, the results for the maximum strain (top) are largely different from each other. Namely, for the DT simulation, a two-step relaxation is seen whereas for PCN the bump is not observed. Although the experiment is difficult, such data would be appreciated for a critical test of the models. Nevertheless, for the small strains (mid and bottom), two simulation results are mostly the same as the conversion factors shown in Equations (1) and (2).
